# Contribution of Levosimendan in Weaning from Mechanical Ventilation in Patients with Left Ventricular Dysfunction: A Pilot Study

**DOI:** 10.1155/2019/7169492

**Published:** 2019-07-24

**Authors:** Ifigeneia Kaltsi, Epameinondas Angelopoulos, Georgios Tzanis, Antonios Sideris, Konstantinos Tyrovolas, Stelios Kokkoris, Christina Gratziou, Serafeim Nanas, Christina Routsi

**Affiliations:** ^1^Intensive Care Unit, Medical School, National and Kapodistrian University of Athens, “Evangelismos” Hospital, Athens, Greece; ^2^Unit of Cardiovascular Interventions, IRCCS San Raffaele Scientific Institute, Milan, Italy; ^3^Coronary Care Unit, Department of Cardiology, “Evangelismos” Hospital, Athens, Greece

## Abstract

**Purpose:**

Mechanically ventilated patients with left ventricular (LV) dysfunction are at risk of weaning failure. We hypothesized that optimization of cardiovascular function might facilitate the weaning process. Therefore, we investigated the efficacy of levosimendan in difficult-to-wean patients with impaired LV performance.

**Materials and Methods:**

Nineteen mechanically ventilated patients, with LV ejection fraction (LVEF) 34 ± 8%, difficult-to-wean from the ventilator, were assessed by transthoracic echocardiography before the start and at the end of a spontaneous breathing trial (SBT) (first SBT). Eight patients successfully weaned. The remaining 11 failed-to-wean patients received a 24-hour infusion of levosimendan, and they were reassessed during a second SBT.

**Results:**

After levosimendan administration, LVEF increased from 30 ± 10 to 36 ± 3% (*p*=0.01). End-SBT peak *e*′ velocity increased from 7 to 9 cm/s (*p*=0.02). *E/e*′ increased from 10.5 to 12.9 during the first SBT, whereas it remained constant at 10 throughout the second SBT (*p*=0.01). During the second SBT, partial pressure of arterial oxygen and central venous oxygen saturation improved, compared to the first one (93 ± 34 vs. 67 ± 28 mmHg, *p*=0.03, and 66 ± 11% vs. 57 ± 9%, *p*=0.02, respectively). Nine of the 11 patients were successfully weaned from the ventilator.

**Conclusions:**

In difficult-to-wean from mechanical ventilation patients with LV dysfunction, levosimendan might contribute to successful weaning by improving both systolic and diastolic LV function.

## 1. Introduction

Among the multiple causes of weaning failure from mechanical ventilation [1], cardiovascular dysfunction has been increasingly recognized as a precipitating factor [2–5]. Responsible pathophysiological mechanisms involve the hemodynamic changes that occur during transition from mechanical ventilation to spontaneous breathing, i.e., from positive to negative intrathoracic pressures. They mainly include an immediate increase of left ventricular (LV) preload and afterload, as well as a decrease of LV compliance [3, 4, 6–8]. Critically ill patients with either overt or occult cardiopulmonary compromise are at significant risk of weaning failure. Particularly, the presence of LV systolic [9–11] and/or diastolic [12–15] dysfunction is deemed a common cause of cardiovascular etiology for weaning failure.

The concept of optimizing the cardiac function before a spontaneous breathing trial (SBT) in anticipation of a difficult weaning process in patients with LV systolic dysfunction is appealing. Still, the administration of inotropes such as dobutamine does not seem to be a reasonable therapeutic option because of the potential adverse effects such as increased heart rate with concomitant-increased myocardial oxygen demand. Furthermore, the sympathetic nervous system activation during failed SBTs [2, 16] does not favor exogenous catecholamine administration. Levosimendan is a calcium sensitizer and an ATP-dependent potassium channels opener that enhances cardiac contractility via positive inotropic and systemic vasodilatory actions, and, unlike dobutamine, it does not increase the myocardial oxygen demand. It is indicated for decompensated heart failure patients with compromised LV function [17–19]. In critical care practice, levosimendan has been mainly used for treating postoperative low cardiac output syndrome in patients undergoing cardiac surgery [20, 21], as well as in weaning from peripheral venoarterial extracorporeal membrane oxygenation [22]. There is limited experience with its use in patients with systolic LV dysfunction, difficult-to-wean from mechanical ventilation [23–25].

We hypothesized that levosimendan would facilitate the weaning process in patients with LV systolic dysfunction and repeatedly failed weaning attempts from mechanical ventilation. To this aim, we studied the efficacy of levosimendan administration in these patients during weaning trials, assessed by transthoracic echocardiography (TTE).

## 2. Materials and Methods

This prospective study was conducted in the multidisciplinary intensive care unit and in the coronary care unit of Evangelismos Hospital, a tertiary referral center. The study was approved by the Hospital Ethics Committee (protocol number 67/6.3.12), and informed consent was obtained from each patient or the patient's next of kin.

### 2.1. Patients

Critically ill patients, mechanically ventilated because of acute respiratory failure of various etiologies, who had difficult or prolonged weaning from mechanical ventilation and LV systolic dysfunction, defined as LV ejection fraction (LVEF) of less than 50% [26], were considered eligible for the study. Difficult weaning was defined as that requiring up to 3 spontaneous breathing trials (SBTs) or as long as 7 days, and prolonged one, that requiring more than 3 SBTs or longer than 7 days [27]. All patients were on diuretic and other established treatments by their attending physicians. Exclusion criteria were ineligibility for echocardiographic study, i.e., poor echocardiographic window, heart rate faster than 110 beats per minute or no sinus rhythm, atrioventricular conduction abnormalities, paced rhythm, significant valvulopathies that can interfere with the Doppler measurements [28], and the absence of an arterial line or a central venous catheter in an internal jugular or subclavian vein.

### 2.2. Respiratory Parameters

Minute ventilation (*V*_E_) was measured at 2 minutes after disconnection from the ventilator with a calibrated Wright spirometer (nSpire Health Ltd., UK) through the endotracheal tube. The average tidal volume (*V*_T_) was obtained by dividing *V*_E_ by the respiratory frequency (*f*). The ratio of *f* to *V*_T_ (*f*/*V*_T_ index) was calculated [29]. Maximum inspiratory pressure was measured as the maximal negative excursion in airway pressure during a 20-second period using a one-way valve [30].

### 2.3. Hemodynamic and Gas Exchange Parameters

Invasive systemic blood pressure, heart rate, and oxygen saturation by using a pulse oximeter (SpO_2_) were continuously monitored. For gas exchange measurements, partial pressures of oxygen (PaO_2_), carbon dioxide (PaCO_2_), pH, and hemoglobin oxygen saturation (SaO_2_) were determined from blood samples drawn from the arterial line and also simultaneously from the internal jugular or the subclavian vein for central venous hemoglobin oxygen saturation (ScvO_2_) measurement, used as a surrogate for the mixed venous oxygen hemoglobin saturation. Samples were immediately analyzed (ABL 600; Radiometer Medical Aps, Brønshøj, Denmark).

### 2.4. TTE Examination

A comprehensive 2-dimensional (2D) and Doppler echocardiography examination was performed in all patients. LV size and function were measured according to the American Society of Echocardiography and European Association of Cardiovascular Imaging recommendations [31, 32]. The 2D echocardiographic study included the evaluation of LV systolic function and left chamber quantification. LVEF was estimated by using the modified Simpson's method in the apical 4-chamber view. The assessment of diastolic function was performed using spectral Doppler parameters [31]. Pulsed-wave Doppler-derived transmitral inﬂow velocities were obtained in the apical 4-chamber view, with the sample volume placed at the level of the mitral valve leaflet tips during maximal opening in diastole. Main diastolic function measurements included the transmitral early diastolic filling (*E*-wave, at early diastole) and late filling (*A*-wave, at late diastole) velocities and the mitral *E/A* ratio (mitral *E*-wave velocity divided by *A*-wave velocity). The deceleration time of the *E*-wave was also measured (DT). Tissue Doppler recordings were measured with sample volume at the lateral mitral annulus. From tissue Doppler imaging, the mitral annulus velocity (*e*′) was obtained with the sample volume placed at the lateral side of the mitral annulus. The ratio of early diastolic LV inflow velocity to lateral mitral annular velocity (*E/e*′) was calculated as an estimate of LV filling pressure. The ratio of peak *E* to peak *e*′ was calculated from the average of at least 3 cardiac cycles.

LV diastolic dysfunction was graded according to the standard recommendations [31]. Patients were classified as having normal diastolic function, grade I or grade II or grade III diastolic dysfunction. Two echocardiographic devices were used for all studies (Vivid 3 and 7, General Electric Healthcare, Milwaukee, WI, USA).

### 2.5. Study Protocol

Before the start of the SBT, all patients were under mechanical ventilation (Maquet Servo I ventilator, Maquet, Sweden), in the assist control mode with the ventilator settings prescribed by the attending physicians. Demographic and clinical characteristics, laboratory values, and fluid balance in the 48 hours prior to SBT were also recorded.

All patients then underwent a SBT via a T-piece (“first SBT”). The SBT lasted for 2 hours, unless patients met any of the criteria for SBT failure: tachypnea (*f* greater than 35 breaths per minute), SpO_2_ less than 90%, tachycardia (heart rate greater than 120 beats per minute) or a sustained change in the heart rate of more than 20%, systolic arterial blood pressure of greater than 180 to 200 mmHg or less than 90 mmHg, arrhythmias, increased accessory muscle use, diaphoresis, and onset or worsening of discomfort [27]. Patients who met these criteria were reconnected to the ventilator, and the SBT was defined as failed, whereas the ability of the patient to remain free of these criteria at the end of the trial was defined as successful SBT and the patient was extubated.

Hemodynamic and gas exchange parameters were recorded immediately before disconnection from the ventilator and at the end of SBTs. TTE examinations were performed at the same time points by cardiologists who were part of the research group, not involved in the management of the patients' weaning process. The decision for the extubation or reinstitution of mechanical ventilation was taken by the physicians responsible for the patients, who were blinded to the TTE results, whereas blood gas values and routine respiratory measurements were available to them.

Subsequently, patients who failed the first SBT were treated with levosimendan (Simdax® 2.5 mg/mL, Orion Pharma), administered by continuous intravenous infusion over 24 hours, at a dose titrated to 0.1-0.2 *μ*g/kg/min, according to hemodynamic tolerance during infusion. A loading dose was not administered to minimize the risk of vasodilation. No other disconnection from the ventilator was attempted during this period, and no other medication was added to their treatment. A repeated SBT was attempted (“second SBT”) within 24 hours after levosimendan infusion.

### 2.6. Statistical Analysis

Continuous variables are presented as mean ± standard deviation or range (median) for heavily skewed distributions, and categorical variables as percentages. We compared the baseline values of patients who failed the first SBT with the values of the patients who successfully completed it using the independent samples *t*-test, the Mann–Whitney *U* test, or Fisher's exact test, as appropriate. We compared the measurements of the group that received levosimendan at the end of the first SBT to those at the end of the second SBT using the paired *t*-test. Analyses were performed with IBM SPSS version 20.

## 3. Results

Nineteen consecutive patients (mean age of 73 ± 8 years, 12 males) were enrolled. Admission diagnosis was surgical in 6 and medical in the remaining 13 patients. Patients had been on mechanical ventilation 11 days (median value, range 7–56 days) and had difficult weaning with repeatedly failed weaning trials. During the first SBT, 8 patients tolerated the trial and were extubated. The remaining 11 patients met the a priori criteria for weaning failure, and mechanical ventilation was reinstituted. Characteristics of the patients according to the first SBT outcome are shown in Table 1; echocardiographic measurements are shown in Table 2.

Compared to those successfully weaned, patients who failed had significantly worse values of MIP (−32 ± (−10) vs. −46 ± (−13) cm H_2_O, respectively, *p*=0.02). There were no significant differences in gas exchange parameters between the two groups. In both groups, baseline ScvO_2_ values were barely normal (66 ± 8% and 66 ± 7%, respectively). Also, proBNP displayed greater variability in the failed trial group.

Comorbidities were evenly distributed with the significant exception of the preadmission diagnosis of systolic heart failure, which every one of the 11 patients in the failed group had. In contrast, among the 8 patients who were successfully weaned, only 4 (50%) had an established diagnosis of systolic heart failure prior to their current admission (Table 1). The remaining 4 patients, though they did not have a known history of heart failure, were diagnosed in the ICU with systolic dysfunction possibly unmasked by critical illness and multiple comorbidities.

Systolic function did not differ significantly between the 2 groups although there was a trend for a lower LVEF for the group of patients who failed to wean from the ventilator. In addition, all patients exhibited some degree of diastolic dysfunction (Table 2).

Among the 11 patients that failed to wean with the first SBT attempt and received levosimendan, 9 patients were successfully extubated after the second SBT. The remaining two patients, one with baseline LVEF = 32% and one with baseline LVEF = 36%, both with grade II diastolic dysfunction, failed the second SBT.

In this subset of 11 patients, *f* was 33 ± 8 and 25 ± 6 breaths/minute at the first and the second SBT, respectively (*p*=0.04). Correspondingly, the *f*/*V*_T_ index dropped from 94 ± 24 at the 2-minute landmark of the first SBT to 69 ± 26 at the same time point of the second SBT (*p*=0.05). PaO_2_ improved from 67 ± 28 mmHg at the end of the first SBT to 93 ± 34 mmHg at the end of the second SBT (*p*=0.03). ScvO_2_ dropped by 6 percentage points during the first SBT and, while it was also reduced during the second SBT, the mean reduction was of 1.8 percentage points. As a result, ScvO_2_ at the end of the second SBT was 66 ± 11% from 57 ± 9% at the end of the first SBT (*p*=0.02).

**Figure 1:**
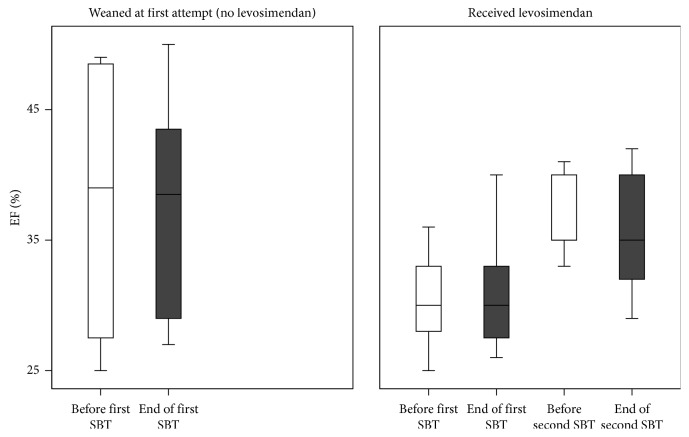
Left ventricular ejection fraction (LVEF) before the start (white boxes) and at the end (grey boxes) of spontaneous weaning trials in successfully weaned patients (a) and in those who failed and received levosimendan (b). After levosimendan administration, LVEF increased.

**Figure 2:**
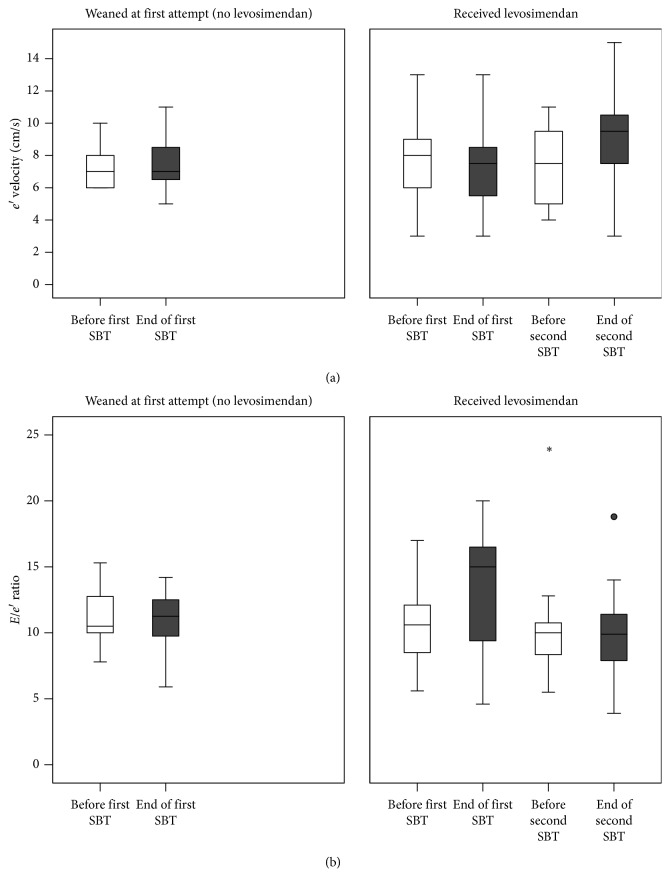
Tissue Doppler *e*′ wave (a) and *E/e*′ ratio (b) before (white boxes) and at the end (grey boxes) of SBTs in successfully weaned patients (left panel) and in those who failed and received levosimendan (right panel). After levosimendan administration, end-SBT *e*′ velocity increased and the *E/e*′ ratio was maintained constant throughout the SBT, whereas it had increased during the first SBT.

The echocardiographic indices at the end of the two SBTs were modified after the treatment, as shown in Table 3. LVEF increased from 30% to 36% (*p*=0.01) between the first and second SBT (Figure 1). End-trial *e*′ velocity signals also increased between the two SBTs, from 7 to 9 cm/s (*p*=0.02, Figure 2(a)). The *E/e*′ ratio, which had increased from a mean of 10.5 to a mean of 12.9 during the first SBT, was maintained relatively constant throughout the second SBT, with a significant decrease in end-SBT values, from 12.8 ± 5.3 after the first to 9.9 ± 4.1 after the second SBT (*p*=0.01). The behavior of *E/e*′ during the second SBT was similar to that observed in the successful group during the first SBT (Figure 2(b)). The *E/A* ratio, on the other hand, remained more or less constant throughout both SBTs, with a mean value of 1.29 at the end of the first SBT and 0.89 at the end of the second SBT. Deceleration time at the end of the second SBT showed a moderate increase to 197 ± 22 ms from 176 ± 33 ms in the first SBT (*p*=0.08).

## 4. Discussion

The present study assessed the effects of levosimendan on LV performance and weaning outcome from mechanical ventilation in patients with LV systolic dysfunction and failed weaning attempts. The main findings are the following: levosimendan administration resulted in (i) a significant increase in LVEF; (ii) maintenance of a constant *E/e*′ throughout the second SBT to significantly lower levels than during the first SBT, associated with a significant increase in *e*′ during the SBT; and (iii) a successful weaning outcome in 9 out of the 11 patients who received the drug.

LV systolic dysfunction is a known risk factor for weaning failure [9–11]. At the beginning of the first SBT, patients who failed, compared to those who were successfully weaned, exhibited at baseline a lower LVEF though the difference was not statistically significant. Nevertheless, those failed-to-wean patients who subsequently received levosimendan exhibited a significant increase in LVEF at the second SBT, indicating a beneficial effect of levosimendan on the LV systolic function.

As tissue Doppler imaging-derived parameter *E/e*′ is considered a reliable surrogate of LV filling pressures [31, 33–36], the absence of *E/e*′ increase during the second SBT in contrast to that observed during the first SBT could possibly indicate that levosimendan reversed the consequences of SBT on LV filling pressures and, accordingly, prevented a potential pulmonary congestion. Moreover, the increase in the early diastolic mitral annulus velocity (*e*′) during the second SBT, in contrast to the first SBT, indicates the left ventricle relaxation rate enhancement [31]. Therefore, as a whole, levosimendan administration resulted in improvement of both systolic and diastolic LV function.

These echocardiographic changes following levosimendan administration were associated with a lesser extent of both PaO_2_ and ScvO_2_ decrease during the second SBT, as compared to the first one, further supporting a possible reduction of weaning-induced increase of LV filling pressures.

The beneficial effects of levosimendan on our patients are consistent with the pharmacological properties of this widely used calcium sensitizer. Specifically, levosimendan increases myocardial contractility by increasing myofilament sensitivity to calcium and is also a potent vasodilator due to activation of ATP-dependent potassium channels in vascular smooth muscle cells, leading to a decrease in RV afterload through pulmonary vasodilatation as well as decreases in both LV preload and afterload. Furthermore, levosimendan has favorable effects on LV diastolic function [37, 38]. Of note, among difficult-to-wean patients, most of the echocardiographic studies have emphasized the common occurrence of LV diastolic, rather than systolic, dysfunction [12–15, 33, 39].

So far, only two studies have evaluated the impact of levosimendan on ventilator-dependent patients with cardiovascular dysfunction [23, 25]. The first study, by Sterba et al. [23], included 12 difficult-to-wean patients with LVEF <40%. Following a 24-hour infusion of levosimendan, LVEF and oxygenation variables significantly improved, contributing to successful weaning in 7 out of 12 patients. The second study, conducted by Ouanes-Besbes et al. [25], assessed the short-term hemodynamic effects of levosimendan as compared to dobutamine, by pulmonary artery catheterization, in 10 difficult-to-wean patients with chronic obstructive pulmonary disease (COPD) experiencing weaning difficulties, associated with increased LV filling pressures. Both drugs reduced the magnitude of pulmonary artery occlusion pressure (PAOP) increase during the SBT. Compared to dobutamine, levosimendan resulted in significantly greater inhibition of SBT-induced increase in PAOP and mean pulmonary artery pressure. Of note, all patients in this study had a LVEF within the normal range. All patients were ultimately extubated without the adjunct of any other cardiovascular drugs.

To our knowledge, the present study is the first to provide precise echocardiographic assessment of the impact of levosimendan upon cardiac function during weaning trials in ventilator-dependent patients with low LVEF, showing beneficial effects in both systolic and diastolic cardiac function and giving, thus, a pathophysiological interpretation for its favorable effects on weaning outcome.

Additionally, use of levosimendan effectively contributed to successful weaning in a patient with dilated cardiomyopathy after repeated weaning failures [24], as well as in another case [40].

### 4.1. Limitations

This study comprised a heterogeneous group of high risk patients for weaning failure, with LV systolic dysfunction and major comorbidities, such as diabetes mellitus, renal impairment, and COPD. The small size of the study and the lack of randomization or a control group are the main limitations that prevent firm conclusions from being drawn regarding the effectiveness of levosimendan on this specific setting. However, each patient was studied four times (both at the start and at the end of two consecutive SBTs, i.e., before and after levosimendan administration), serving, thus, as their own control. A further limitation could be considered that the s' wave was not assessed in order to estimate the systolic function. However, to this end, we used Simpson's method, which is considered the gold standard method of the systolic function estimation.

In conclusion, among mechanically ventilated patients with LV systolic dysfunction and difficult-to-wean from mechanical ventilation, levosimendan administration induced beneficial effects on both systolic and diastolic function and, possibly, contributed to the observed successful weaning from mechanical ventilation. Although pilot, this study might help to a future design of a randomized trial. Probably, further studies are warranted to confirm these results.

## Figures and Tables

**Table 1:** Clinical characteristics of the patients according to the first SBT outcome.

	First SBT (*n*=19)		*p* value
	Successful (*n*=8)	Failed (*n*=11)	
Age (years)	74 ± 8	72 ± 9	0.6
Females (%)	2 (25)	4 (36)	0.9
MIP (cmH_2_O)	−46 ± (−13)	−32 ± (−10)	0.02
Troponin (pg/ml)^*∗*^	432 ± 576	217 ± 268	0.36
NT-proBNP (pg/ml)^*∗*^	1564 ± 862	7515 ± 13643	0.36
Systolic BP (mmHg)^*∗*^	129 ± 25	123 ± 18	0.57
Diastolic BP (mmHg)^*∗*^	63 ± 15	60 ± 15	0.59
Heart rate (bpm)^*∗*^	91 ± 18	83 ± 15	0.31
SaO_2_ (%)^*∗*^	99 ± 1	98 ± 1	0.69
PaO_2_ (mmHg)^*∗*^	125 ± 26	132 ± 40	0.68
PaCO_2_ (mmHg)^*∗*^	36 ± 5	38 ± 10	0.63
pH^*∗*^	7.41 ± 0.02	7.42 ± 0.04	0.69
ScvO_2_ (%)^*∗*^	66 ± 8	66 ± 7	0.9
Hct (%)	25.7 ± 3.7	28.8 ± 4.3	0.1
48-hour fluid balance (mL)^*∗*^	371 ± 1134	618 ± 1646	0.72

*No. of patients with*			
Ischemic heart disease, *n* (%)	4 (50)	9 (81)	0.32
Heart failure, *n* (%)	4 (50)	11 (100)	0.02
Hypertension, *n* (%)	7 (88)	11 (100)	0.42
Atrial fibrillation, *n* (%)	3 (38)	2 (18)	0.62
Diabetes mellitus, *n* (%)	3 (38)	4 (36)	0.90
Chronic kidney disease, *n* (%)	3 (35)	5 (45)	0.90
COPD, *n* (%)	2 (25)	3 (27)	0.90

SBT, spontaneous breathing trial; MIP, maximum inspiratory pressure; BP, blood pressure; NT-proBNP, *N*-terminal probrain natriuretic peptide; bpm, beats per minute; SaO_2_, arterial oxygen saturation; PO_2_, arterial oxygen tension; PaCO_2_, arterial carbon dioxide tension; pH, arterial pH; ScvO_2_, central venous oxygen saturation; Hct, haematocrit; COPD, chronic obstructive pulmonary disease; (^*∗*^), before the start of the first SBT.

**Table 2:** Echocardiographic indices before the start of the first spontaneous breathing trial.

	First spontaneous breathing trial		*p* value
	Successful (*n*=8)	Failed (*n*=11)	
LVEF (%)	38 ± 10	30 ± 4	0.08
*E* velocity (cm/s)	80 ± 15	86 ± 34	0.66
*A* velocity (cm/s)	79 ± 45	74 ± 24	0.79
Deceleration time (ms)	214 ± 60	234 ± 66	0.51
*e*′ velocity (cm/s)	7 ± 1	8 ± 2	0.31
*E/e*′ ratio	11.20 ± 2.32	10.51 ± 3.18	0.61
*E/A* ratio	1.33 ± 0.79	1.14 ± 0.76	0.64

Diastolic dysfunction			
Grade I	2	1	0.48
Grade II	4	9	
Grade III	2	2	

*E*, peak velocity of *E-*wave; *A*, peak velocity of *A*-wave measured using Doppler transmitral flow; *e*′, early mitral annulus diastolic velocity measured using tissue Doppler imaging; LVEF, left ventricular ejection fraction.

**Table 3:** Absolute change in echocardiographic parameters on spontaneous breathing trial (SBT) before and after levosimendan infusion (*n*=11).

		*p* value
Delta LVEF (%)	4.6 ± 4.9	0.01
Delta *E* velocity (cm/s)	−2.0 ± 1.9	0.65
Delta *A* velocity (cm/s)	9.0 ± 2.2	0.27
Delta deceleration time (ms)	21.4 ± 34.4	0.08
Delta *e*′ velocity (cm/s)	2 ± 2	0.02
Delta *E/e*′ ratio	−2.90 ± 3.34	0.01
Delta *E/A* ratio	−0.27 ± 0.59	0.24

Differences were calculated as 2nd – 1st SBT values. *E*, peak velocity of *E-*wave; *A*, peak velocity of *A*-wave measured using Doppler transmitral flow; *e*′ early mitral annulus diastolic velocity measured using tissue Doppler imaging; LVEF, left ventricular ejection fraction.

## Data Availability

The data used to support the findings of this study are available from the corresponding author upon request.

## Abbreviations

LV: Left ventricular
LVEF: Left ventricular ejection fraction
TTE: Transthoracic echocardiography
SBT: Spontaneous breathing trial
*V*_E_: Minute ventilation
*V*_T_: Tidal volume
*f*: Respiratory frequency
SpO_2_: Oxygen saturation by pulse oximeter
SaO_2_: Hemoglobin oxygen saturation
ScvO_2_: Central venous hemoglobin oxygen saturation
PaO_2_: Partial pressure of oxygen
PaCO_2_: Partial pressure of carbon dioxide
MIP: Maximum inspiratory pressure
*E*: Peak velocity of *E-*wave
*A*: Peak velocity of *A*-wave measured using Doppler transmitral flow
*e*′: Early mitral annulus diastolic velocity measured using tissue Doppler imaging.
